# The relation of maternal hypothyroidism and hypothyroxinemia during pregnancy on preterm birth: An updated systematic review and meta-analysis

**Published:** 2017-09

**Authors:** Marzieh Parizad Nasirkandy, Gholamreza Badfar, Masoumeh Shohani, Shoboo Rahmati, Mohammad Hossein YektaKooshali, Shamsi Abbasalizadeh, Ali Soleymani, Milad Azami

**Affiliations:** 1 *Department of Obstetrics and Gynecology, Women’s Reproductive Health Research Center, School of Medicine, Tabriz University of Medical Sciences, Tabriz, Iran.*; 2 *Department of Pediatrics, Behbahan Faculty of Medical Sciences, Ahvaz Jundishapour University of Medical science, Behbahan, Iran.*; 3 *Department of Nursing, Faculty of Allied Medical Sciences, Ilam University of Medical Sciences, Ilam, Iran.*; 4 *Student Research Committee, Ilam University of Medical Sciences, Ilam, Iran.*; 5 *Student Research Committee, School of Nursing- Midwifery, and Paramedicine, Guilan University of Medical Sciences, Rasht, Iran.*; 6 *Faculty of Medicine, Dezful University of Medical Sciences, Dezful, Iran.*

**Keywords:** Hypothyroidism, Pregnancy, Preterm birth, Meta-analysis, Cohort

## Abstract

**Background:**

The clinical consequences of hypothyroidism and hypothyroxinemia during pregnancy such as preterm birth are not still clear.

**Objective:**

The aim of this meta-analysis was to estimate the relation of clinical and subclinical hypothyroidism and hypothyroxinemia during pregnancy and preterm birth.

**Materials and Methods:**

In this meta-analysis, Preferred Reporting Items for Systematic review and Meta-Analysis were utilized. Searching the cohort studies were done by two researchers independently without any restrictions on Scopus, PubMed, Science Direct, Embase, Web of Science, CINAHL, Cochrane, EBSCO and Google Scholar databases up to 2017. The heterogeneity of the studies was checked by the Cochran's Q test and I^2^ index. Both random and fixed-effects models were used for combining the relative risk and 95% confidence intervals. Data were analyzed using Comprehensive Meta-Analysis software version 2.

**Results:**

Twenty-three studies were included in the meta-analysis. The relative risks of the clinical hypothyroidism, subclinical hypothyroidism and hypothyroxinemia during pregnancy on preterm birth was estimated 1.30 (95% CI: 1.05-1.61, p=0.013, involving 20079 cases and 2452817 controls), 1.36 (95% CI: 1.09-1.68, p=0.005, involving 3580 cases and 64885 controls) and 1.31 (95% CI: 1.04-1.66, p=0.020, involving 1078 cases and 44377 controls), respectively.

**Conclusion:**

The incidence of preterm birth was higher among mothers with clinical and subclinical hypothyroidism or hypothyroxinemia during pregnancy compared to euthyroid mothers, and these relations were significant. Therefore, gynecologists and endocrinologists should manage these patients to control the incidence of adverse pregnancy outcomes such as preterm birth.

## Introduction

Thyroid hormones are needed for normal metabolism, regulation of body temperature, energy production, and fetal development (1). Changes in maternal thyroid function during pregnancy and lack of adequate adaptation to these changes will lead to thyroid dysfunction (2, 3). Some of these changes in thyroid function happen due to increased levels of thyroid binding globulin, an increase in renal clearance of iodine and thyrotrophic effect on human chorionic gonadotropin (4, 5). The prevalence of subclinical hypothyroidism during pregnancy is reported 1.5-4% and for clinical hypothyroidism 0.5-3% (6-8). However, the cutoff point, ethnicity, and the research design can be effective in this controversy. But generally, it is more prevalent in Asian countries (8).

In order to achieve the favorable result of pregnancy, which is a full-term and alive baby, all the conditions should be optimized in early pregnancy. Proper thyroid function of the mother, especially in the first trimester for fetus brain development and also when the fetus is not capable of producing thyroid hormones, is critical (9). The clinical consequences of hypofunction thyroid during pregnancy on adverse pregnancy outcomes such as premature birth are controversy (10-14). Systematic review and meta-analysis study by examining all relevant documentation and providing an overall estimate can present a full picture of problem in pregnant women (15, 16). 

Given the importance of these disorders, especially hypothyroidism during pregnancy and also the inconsistent results of different studies in this field, this systematic review and meta-analysis study was conducted with the purpose of assessing the adverse effects of clinical hypothyroidism, subclinical hypothyroidism, and hypothyroxinemia during pregnancy on preterm birth. 

The results obtained in this study could provide valuable information from the findings of multiple studies. It also can be a basis for creating new plans and programs to properly manage these disorders during pregnancy for prevention of preterm birth.

## Material and methods

**Study protocol**

This meta-analysis was done in several detailed stages, including search strategy, determining the inclusion and exclusion criteria, quality evaluation of studies, data extraction, analysis and interpretation of findings by using the preferred reporting items for systematic reviews and meta-analyses protocol (PRISMA-P) (17). In order to avoid error and bias, all procedures were done by two researchers who were independent of each other.

**Search strategy**

Literature searching was done by two researchers independently who were familiar with search methods and information sources without any restrictions on Scopus, PubMed, Science Direct, Embase, Web of Science, CINAHL, Cochrane, EBSCO as well as Google scholar databases up to 2017 using keywords including thyroid disease, thyroid, hypothyroidism, subclinical hypothyroidism, clinical hypothyroidism, hypothyroxinemia, preterm delivery, premature delivery, preterm labor, premature labor, preterm birth and premature birth which being searched in combination using AND & OR operators. Combined search in PubMed is shown in Appendix 1. In order to achieve more studies, review articles and all relevant references were evaluated as well. Also, any encounters discussed by third expert researcher.

**Inclusion and exclusion criteria**

The study was considered to be eligible if the following criteria were met: 1) A prospective cohort study; 2) The mother suffered from clinical or subclinical hypothyroidism or hypothyroxinemia during pregnancy for case group; 3) Preterm birth was investigated in the outcome 4) The mother was not thyroid autoimmunity disease; 5) The mother was not receiving treatment for thyroid hypofunction and 6) Information about the number of preterm births in each generation was reported. In this investigation, data from review articles, case-controls, case reports, and letters to the editor were not reviewed.

**Definitions**

Preterm birth defined as a premature birth in less than 37 gestational weeks. A high thyroid-stimulating hormone (TSH) level with a low free thyroxine (FT4) level; a high TSH level with a normal FT4 level; and a low FT4 level with a normal TSH level was defined as clinical hypothyroidism, subclinical hypothyroidism, and hypothyroxinemia, respectively.

**Quality evaluation**

After determining the relevant investigations, selected papers were evaluated according to the STROBE checklist (18). The checklist consists of 22 sections which evaluate various aspects of the methodology. The researchers chose a simple method for scoring; 0-2 points were given to each question in the checklist, so maximum points attainable was considered to be 44. The papers were divided into three categories in terms of quality: Low (0-15), medium (16-30), and high quality (31-44). The articles that get a minimum score of 16 were gotten into the meta-analysis. 

**Data extraction**

The researchers used a checklist containing the required information for studying the articles, including the author's name, article title, year of study, place of study, sample size, age, gestational age, any information on the incident of maternal thyroid disease, and preterm birth compared to a reference group.

**Statistical analysis**

Relative risk (RR) was used in order to determine the effect size of maternal hypothyroidism and hypothyroxinemia during pregnancy on preterm birth. RR with 95% confidence intervals (CIs) from selected studies was pooled. Heterogeneity among the investigated studies was determined using Cochran's Q test and I^2^ index. There are three categories for heterogeneity (less than 25% or low, between 25-50% considered as moderate, and above 50% as high heterogeneity) (19).

Therefore, in this study fixed-effects and random-effects were performed for low and high heterogeneity, respectively (20). Becuase of high heterogeneity, subgroup analysis based on the continent was performed to find sources of heterogeneity. Cumulative meta-analysis was performed based on the published year of the study to determine the year of acceptance or rejection of assumptions. Sensitivity analysis was conducted to assess the validity and reliability of results, and to show the effect of removing single study on the overall estimate at a time. Egger and Begg’s tests for checking Publication bias was used (21-22). The data was analyzed using meta-analysis specialized software, Comprehensive Meta-Analysis software version 2. The significance level was considered lower than 0.05.

## Results

**Search results**

**Figure 1:**
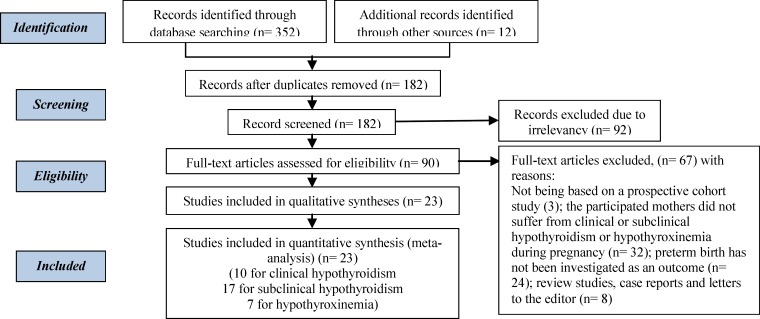
Study selection process

In this systematic search, 364 possible relevant studies were identified After further investigation, 342 studies were removed due to the lack of following criteria: duplication (182); irrelevant (92); not being based on a prospective cohort study (3); the participated mothers did not suffer from clinical or subclinical hypothyroidism or hypothyroxinemia during pregnancy (n=32); preterm birth has not been investigated as an outcome (n=24); review studies, case reports and letters to the editor (n=8) (Figure 1). Finally, 23 qualified studies (10 for clinical, 17 for subclinical hypothyroidism and 7 for hypothyroxinemia) entered into the quantitative meta-analysis (Table I).

**Clinical hypothyroidism**

**Figure 2:**
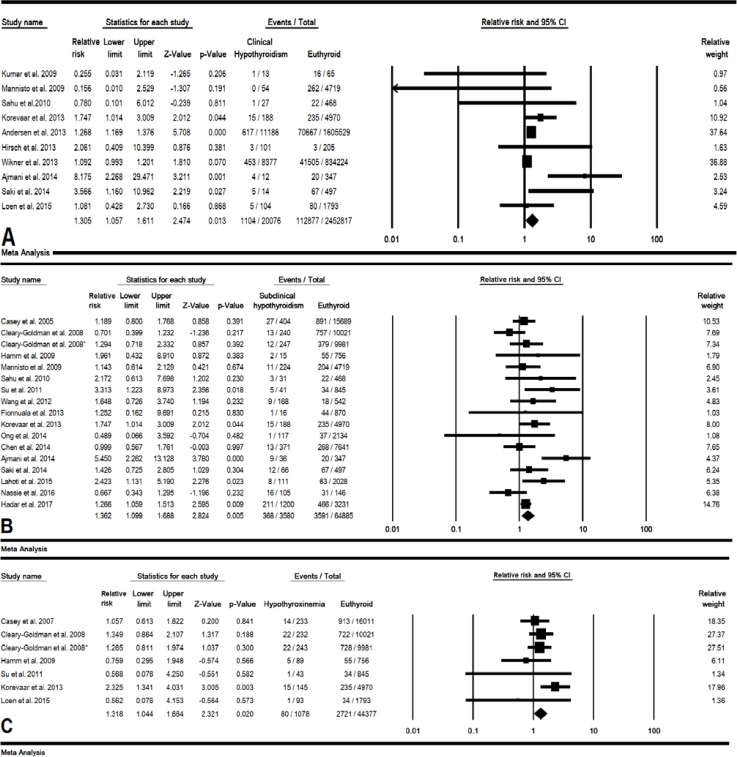
Forest plot for relative risk (RR) in clinical hypothyroidism (A), subclinical hypothyroidism (B) and hypothyroxinemia (C) during pregnancy. For A and B according to High heterogeneity, random effects model and for C according to low heterogeneity, fixed effects model was used

**Figure 3:**
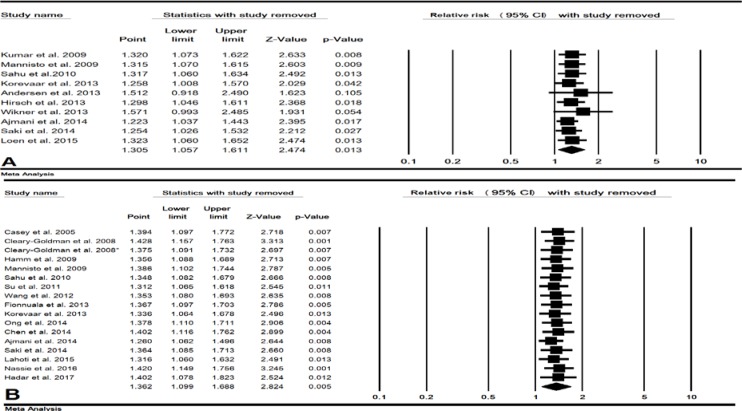
Forest plot for sensitivity analysis in clinical hypothyroidism (A) and subclinical hypothyroidism (B) during pregnancy on preterm.

**Figure 4:**
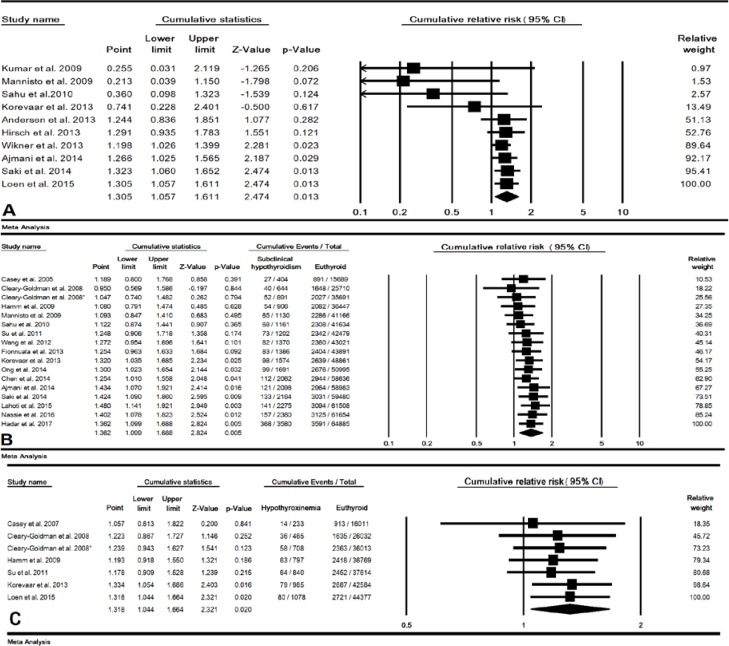
Forest plot for cumulative relative risk (RR) in clinical hypothyroidism (A), subclinical hypothyroidism (B) and hypothyroxinemia (C) during pregnancy on preterm

In 10 studies (20079 cases and 2452817 control pregnant women), the combined RR of maternal clinical hypothyroidism during pregnancy on preterm birth was estimated 1.30 (95% CI: 1.05-1.61, p=0.013) (Figure 2-A). In a sensitivity analysis, after removing single study of Andersen *et al* and Wikner *et al* the total p-value for this relationship increased to 0.105 and 0.054, respectively, indicating the low sensitivity of this meta-analysis (Figure 3-A). The cumulative meta-analysis for this relationship was shown in 2014, this relationship was statistically significant (Figure 4-A). The combined RR for this relationship by continent (Table II), and RR for Asian and European countries was estimated 2.06 (95% CI: 0.70-6.05, p=0.184) and 1.20 (95% CI: 1.03-1.39, p=0.016), respectively.

**Subclinical hypothyroidism**

In 17 studies (3580 cases and 64885 control pregnant women), the combined RR of maternal subclinical hypothyroidism during pregnancy on preterm birth was estimated 1.36 (95% CI: 1.09-1.68, p=0.005) (Figure 2-B). The result of sensitivity analysis for total RR for this relationship was not affected by removing single study which meant this estimate had a good stability (Figure 3-A). The result of cumulative meta-analysis for this relationship was shown in Figure 4-B, the result was indicated in 2013, this relationship was statistically significant. The RR for this relationship in Asian studies was significant (p=0.009) but in American and European studies a significant relationship was not found (p=0.628 and p=0.072 respectively) (Table II).

**Isolated hypothyroxinemia**

In 7 studies (1078 cases and 44377 control pregnant women), the combined RR of maternal hypothyroxinemia during pregnancy on preterm birth was estimated was estimated 1.31 (95% CI: 1.04-1.66, p=0.020), and the association was statistically significant (Figure 2-C). Also, a cumulative meta-analysis was indicated in 2013, this relationship was statistically significant.

**Publication bias**

The Result for Egger test in clinical hypothyroidism (0.57), subclinical hypothyroidism (0.37) and hypothyroxinemia (0.22) was estimated. The Result for Begg’s test in clinical hypothyroidism (0.21), subclinical hypothyroidism (0.23) and hypothyroxinemia (0.76) was estimated. Publication bias in the obtained results is shown in Figure 4 which shows as symmetrical in Funnel Plot.

**Table I.** Basic characteristics of included cohort studies for A) clinical hypothyroidism, B) subclinical hypothyroidism, and C) hypothyroxinemia

**First author**	**Year**			**Country**			**Case (n)**					**Control (n)**	**Follow-up (yr)**			**Gestational age (wk)**	**RR for ** **PB**	**(95%CI)**		
																		**Lower **		**Upper **
A																				
Korevaar *et al* (10)		2013			Netherlands			188		4970			-		<18		1.74		1.01	3.00
Ajmani *et al* (11)		2014			India			12		347			1		13-26		8.17		2.26	29.47
Andersen *et al* (12)		2013			Denmark			11186		1605529			11		Before, during, and after pregnancy		1.26		1.16	1.37
Loen *et al* (13)		2015			Spain			104		1793			11		<13		1.08		0.42	2.73
Wikner *et al* (14)		2013			Sweden			8377		834224			1		<13		1.09		0.99	1.20
Sahu *et al* (23)		2010			India			27		468			9		13-26		0.78		0.10	6.01
Kumar *et al* (24)		2009			India			13		65			3		5-39		0.25		0.031	2.11
Saki *et al* (25)		2014			Iran			14		497			1		15-28		3.56		1.16	10.96
Hirsh *et al* (26)		2013			Israel			34		92			3		29-41		2.06		0.40	10.33
Mannisto *et al* (27)		2009			Finland			54		4719			6		≥23		0.15		0.01	2.52
B																				
Wang *et al* (28)		2012			China			168		542			3		≤12		3.35		1.3	3.75
Cleary-Goldman *et al* (29)		2008			USA			240		10021			3		First trimester		0.70		0.39	1.23
Cleary-Goldman *et al* (29)		2008			USA			247		9981			3		Second trimester		1.29		0.7	2.33
Korevaar *et al* (10)		2013			Netherlands			188		4970			-		<18		1.74		1.01	3.00
Casey *et al*(30)		2005			USA			404		15689			4		<20		1.18		0.8	1.76
Su *et al* (31)		2011			China			41		845			3		<20		3.31		1.22	8.97
Mannisto *et al* (27)		2009			Finland			224		4719			2		<20		1.14		0.61	2.12
Ajmani *et al* (11)		2014			India			36		347			1		13-26		5.45		2.26	13.12
Ong *et al *(32)		2014			Australia			117		2134			1		9-14		0.48		0.06	3.59
Chen *et al* (33)		2014			China			371		7641			2		All trimester		0.99		0.56	1.76
Sahu *et al* (23)		2010			India			31		468			3		13-26		2.17		0.61	7.69
Saki *et al* (25)		2014			Iran			14		497			1		15-28		1.42		0.72	2.80
Lahoti *et al* (34)		2015			India			111		2028			3		All trimester		2.42		1.13	5.19
Nassie *et al*(35)		2016			Israel			105		146			-		23-34		0.66		0.34	1.29
Hadar *et al*(36)		2017			Israel			1200		3231			5		First trimester		1.26		1.05	1.51
Fionnuala *et al* (37)		2013			Ireland			16		870			-		Second trimester		1.25		0.16	9.69
Hamm *et al* (38)		2009			Canada			89		759			1		15-16		1.96		0.043	8.91
C																				
Cleary-Goldman *et al* (29)			2008			USA			232		10021			3	First trimester		1.15		0.72	1.84
Cleary-Goldman *et al* (29)			2008			USA			243		9981			3	Second trimester		1.2		0.77	1.88
Korevaar *et al* (10)			2013			Netherlands			145		4970			-	<18		2.54		1.42	4.54
Hamm *et al* (38)			2009			Canada			89		756			1	15-16		0.79		0.38	1.67
Loen *et al* (13)			2015			Spain			93		1793			11	<13		0.92		0.28	3.01
Su *et al* (31)			2011			China			43		845			3	<20		0.56		0.07	4.25
Casey *et al* (39)			2007			USA			233		16011			4	<20		1.05		0.61	1.82

RR: Relative Risk

CI: Confidence Interval

PB: Preterm birth

**Table II:** Subgroup analysis based on the continent for relative risk (RR) in A) clinical hypothyroidism and B) subclinical hypothyroidism during pregnancy on preterm

**Variable**		**No. of studies**	**Sample size(N)**		**Heterogeneity**		**95% CI**	**RR**	**p-value**
			**Case (n)**	**Control (n)**	**p-value**	**I** ^2^ ** (%)**			
Clinical hypothyroidism									
	Asia	5	167	1582	0.052	57.44	0.70-6.05	2.06	0.184
	Europe	5	19909	2451235	0.051	57.69	1.03-1.39	1.20	0.016
Test for subgroup differences: P=0.324									
Subclinical hypothyroidism									
	Asia	9	2129	15745	0.005	63.26	1.12-2.29	1.60	0.009
	Australia	1	117	2134	-	0	0.06-3.59	0.48	0.482
	Europe	3	428	10559	0.597	0	0.96-2.15	1.44	0.072
	USA	4	906	36447	0.328	12.90	0.79-1.47	1.08	0.628
Test for subgroup differences: P=0.269									

CI: Confidence Interval

RR: Relative Risk

**Figure 5:**
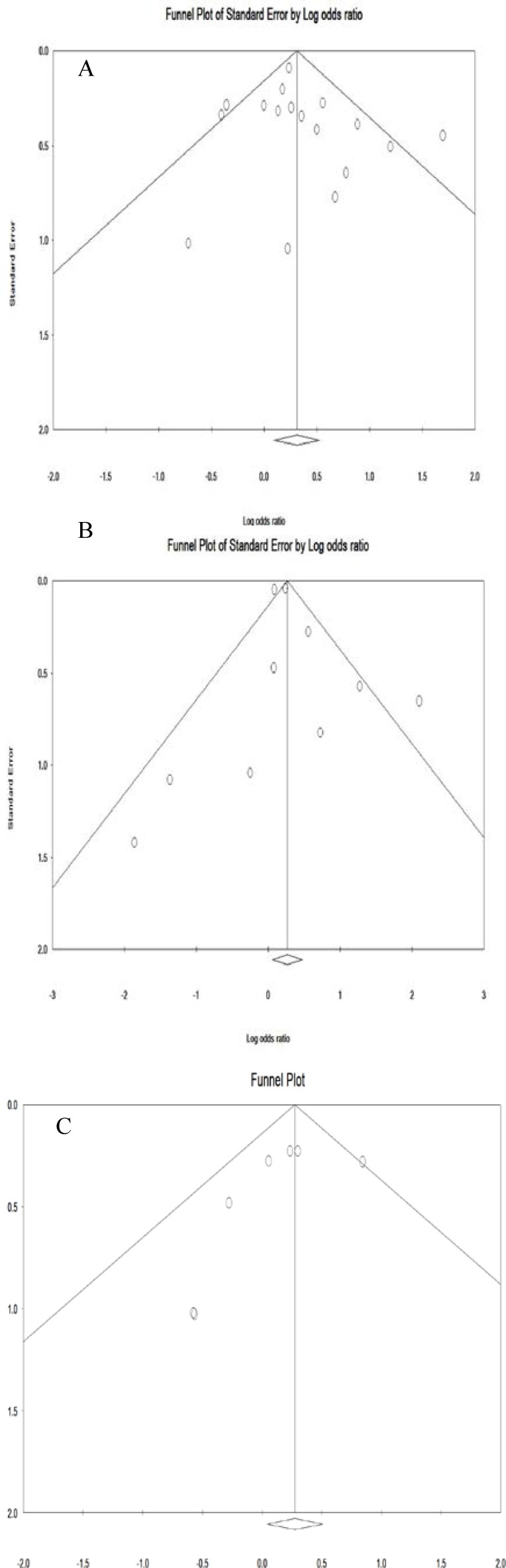
Publications bias of included studies for clinical hypothyroidism (A), subclinical hypothyroidism (B) and hypothyroxinemia (C) during pregnancy on preterm

## Discussion

In the present meta-analysis, the combined 17, 10 and 7 studies for subclinical hypothyroidism, clinical hypothyroidism and hypothyroxinemia during pregnancy showed that are related to preterm birth, with p-values of, 0.013, 0.005, and 0.020, respectively. The mechanism that hypothyroidism can increase the risk of premature birth may be affected by different paths. One possible explanation is that inflammatory process with a change in the regulation of cytokine networks in the uterus and omission of the pair-control inflammatory processes can be linked with premature birth (40-42). Another suggestion are that thyroid hormones may influence fetal development directly through action on maternal and fetal metabolism (43).

The findings of one meta-analysis study on maternal thyroid dysfunction and its impact on pregnancy with combining only two studies concluded that there is no relationship between maternal thyroid disorder and unpleasant pregnancy outcomes (9). According to another meta-analysis, performed by Sheehan et al. on 6 studies and Hou et al. on 6 other studies, there is a significant relationship between clinical hypothyroidism and preterm birth (44, 45). Maraka et al. in their meta-analysis of 14 studies on the association between subclinical hypothyroidism in pregnant women with the incidence of preterm birth, indicated no association between maternal subclinical hypothyroidism and risk of preterm birth (46). In another meta-analysis study by Sheehan combining 10 studies, this relationship was not significant (p=0.32) (40). Also, Nazarpour *et al*. in a systematic review study suggested that further studies on neonatal outcomes of maternal subclinical hypothyroidism are essential (47). For hypothyroxinemia and preterm birth, the combined RR was 1.31 and the association was significant. In Sheehan et al. meta-analysis of 4 studies, this association was not significant (44). Hypothyroxinemia is a controversial management problem during pregnancy which can reassure practitioners that pregnant women with hypothyroxinemia have safety approach. It was revealed in a study that treatment of hypothyroxinemia in order to maintain FT4 above the normal top range may prevent preterm birth in multiparous women (48). 

However, one important limitation of their studies compared to the present study was the lack of studies and less sample size, which can affect the results of the analysis. The results of the meta-analysis are reliable when all the investigations get through the quantitative analysis process which leads to lower variations and possibilities (16-17).

## Conclusion

The incidence of preterm birth was higher among mothers with clinical hypothyroidism or subclinical hypothyroidism or hypothyroxinemia during pregnancy compared to euthyroid mothers, and these relations were significant. Therefore, gynecologists and endocrinologists should manage these patients to control the incidence of adverse pregnancy outcomes such as preterm birth. In future studies, clinical trial studies in the field of subclinical and clinical hypothyroidism, as well as the role of various treatments to prevent premature birth, is recommended.
